# *CaCP15* Gene Negatively Regulates Salt and Osmotic Stress Responses in *Capsicum annuum* L.

**DOI:** 10.3390/genes14071409

**Published:** 2023-07-07

**Authors:** Luyao Zhou, Sizhen Yang, Chunlin Chen, Meng Li, Qingjie Du, Jiqing Wang, Yanxu Yin, Huaijuan Xiao

**Affiliations:** 1 Department of Horticulture, Henan Agricultural University, Zhengzhou 450002, China; 18860363378@163.com (L.Z.);; 2 Cash Crops Research Institute, Hubei Academy of Agricultural Sciences, Wuhan 430064, China

**Keywords:** pepper, *CaCP15* gene, expression analysis, functional analysis

## Abstract

Salt and osmotic stress seriously restrict the growth, development, and productivity of horticultural crops in the greenhouse. The papain-like cysteine proteases (PLCPs) participate in multi-stress responses in plants. We previously demonstrated that salt and osmotic stress affect cysteine protease 15 of pepper (*Capsicum annuum* L.) (*CaCP15*); however, the role of *CaCP15* in salt and osmotic stress responses is unknown. Here, the function of *CaCP15* in regulating pepper salt and osmotic stress resistance was explored. Pepper plants were subjected to abiotic (sodium chloride, mannitol, salicylic acid, ethrel, methyl jasmonate, etc.) and biotic stress (*Phytophthora capsici* inoculation). The *CaCP15* was silenced through the virus-induced gene silencing (VIGS) and transiently overexpressed in pepper plants. The full-length *CaCP15* fragment is 1568 bp, with an open reading frame of 1032 bp, encoding a 343 amino acid protein. CaCP15 is a senescence-associated gene 12 (SAG12) subfamily member containing two highly conserved domains, Inhibitor 129 and Peptidase_C1. *CaCP15* expression was the highest in the stems of pepper plants. The expression was induced by salicylic acid, ethrel, methyl jasmonate, and was infected by *Phytophthora capsici* inoculation. Furthermore, *CaCP15* was upregulated under salt and osmotic stress, and *CaCP15* silencing in pepper enhanced salt and mannitol stress resistance. Conversely, transient overexpression of *CaCP15* increased the sensitivity to salt and osmotic stress by reducing the antioxidant enzyme activities and negatively regulating the stress-related genes. This study indicates that *CaCP15* negatively regulates salt and osmotic stress resistance in pepper via the ROS-scavenging.

## 1. Introduction

The growth, development, and yield of crops are seriously affected by various environmental stresses, such as drought, salt, osmotic, heat, cold, UV radiation, heavy metals, pathogenic bacteria, etc. Drought and salinity are two primary abiotic stresses affecting crop yields globally [1]. They also cause secondary salinization of greenhouse soil, limiting the growth of horticultural crops [2]. The suitable prevention strategy is cultivating high-yield and abiotic stress-resistant crops aided by molecular genetics. Molecular genetics is important in determining pivotal genes and regulatory modules involved in salt and drought tolerance and adaptability of stress-tolerant crop plants [3,4]. Plants activate physiological, morphological, and biochemical processes in response to the changing environment [5,6]. Abiotic stresses enhance the accumulation of reactive oxygen species (ROS) and H_2_O_2_ (hydrogen peroxide) in peroxisomes, mitochondria, chloroplasts, and other organelles. ROS production is very common in plants under different stress conditions [7]. ROS causes oxidative stress in plant cells, damaging lipids, metabolites, proteins, and nucleic acids, thus affecting multiple biological processes [8]. Proteases rapidly degrade the damaged proteins through proteolysis, which is necessary to regulate stress-signaling molecules by clearing misfolded or unwanted proteins. The cysteine proteases (CPs) play important roles in the proteolysis process of higher plants [9].

Cysteine proteases are an indispensable plant protease family which plays dynamic roles in plant growth and development through proteolysis [10]. Salinity stress increases ROS accumulation and the resulting damaged proteins in cells. Therefore, degrading these proteins is essential for improved plant growth and development during salt stress. Plant proteases participate in salt stress responses, and cysteine proteases play considerable roles in degrading oxidized proteins and regulating ROS contents [11]. The transcriptional level of RD21a and RD19a cysteine proteases were increased under salt and dehydration stress [12]. The mRNA levels of the *Cyp15a* gene were increased in peas (*Pisum sativum*) in response to high salt stress [13]. Furthermore, the *SPCP2* gene altered the salt and drought stress tolerance of sweet potatoes [14]. Thus, cysteine proteases regulate physiological processes and the signaling pathway of salt stress responses. Hence, identifying the various stress-related proteases and their function may provide more information for increasing the stress resistance and yield potentials of crops.

Our previous study showed the PLCPs family in pepper (*Capsicum annuum* L.) is divided into SAG12, RD21, XCP, CEP, XBCP3, THI, RD19, ALP, and CTB subfamilies. CaCP15 is a member of the SAG12 (senescence-associated gene 12) subfamily, the largest and richly functional. AtSAG12 is involved in nitrogen remobilization of seed filling and yields [15]. SAG12 is closely associated with senescence and results in high hexose contents in senescent *Arabidopsis* leaves [16,17,18,19]. NtSAG12 is responsible for amino acid remobilization in tobacco, while OsSAG12 protease negatively regulates stress-induced cell death in rice [20,21]. 

This study explored how *CaCP15* regulates salt and osmotic stress. We first analyzed the molecular characteristic of *CaCP15* and investigated its transcriptional level under abiotic and biotic stress. Virus-induced gene silencing (VIGS) of *CaCP15* increased salt and osmotic tolerance in pepper; however, *CaCP15* overexpression reduced the salt and osmotic tolerance in pepper. *CaCP15*-overexpressing pepper scavenged ROS via the antioxidant enzymes, and altered the transcriptional levels of the stress-related genes under salt and osmotic stress. Collectively, these results implied that *CaCP15* potentially induced salt and osmotic tolerance by co-regulating the antioxidant defense enzymes in pepper.

## 2. Material and Methods

### 2.1. Plant Material and Treatments

The seedlings of pepper cultivar B12 were grown under 25/21 °C 16/8 h day/night in a growth chamber with 60% of relative humidity. After growing the 6–8 true leaves stage, the seedlings were subjected to different stress-inducing treatments (100 mM abscisic acid (ABA), 1 mM methyl jasmonate (MeJA), 5 mM salicylic acid (SA), 1 mM ethylene (ETH), 300 mM sodium chloride (NaCl), 300 mM mannitol, 40 °C, 4 °C, and 100 mM hydrogen peroxide (H_2_O_2_))[22]. The second to fourth true leaves were sampled at 0, 3, 6, 12, 24, and 48 h. The qRT-PCR was conducted for the tissue-specific analysis of *CaCP15* using roots, stems, young leaves, mature leaves, old leaves, flower buds, flowers, and fruits (green and red fruits) [22]. For the fungal pathogen stress, the stem base of the plants was inoculated with *Phytophthora capsici* (*P*. *capsici*) mycelia-containing agar discs, and the plants were placed in an artificial climate chamber at 28 °C, with 16/8 h light/dark photoperiod and relative humidity of 80%. Leaf samples were collected at intervals of 0, 3, 6, 12, 24, 48, 72, and 96 h.

### 2.2. RNA Isolation and qRT-PCR Analysis

Total RNA was extracted from tissues and leaves of pepper plants under different stress treatments using Trizol (Invitrogen, Carlsbad, CA, USA) method. Complementary DNA (cDNA) was synthesized using the PrimeScript™Kit (TaKaRa, Tokyo, Japan) reagent. The cDNA concentration was measured using a NanoDrop instrument (UNano 1000F, Hangzhou, China) and normalized to 50 ng/ul. The qRT-PCR tests were performed using SYBR^®^Premix Ex Taq™II (TaKaRa) reagents, and capsaicin ubiquitin-coupled protein gene (*CaUBI3*) (accession number: AY486137.1) was used as the reference gene. The experiment was conducted in triplicate, and the relative expression levels of genes were calculated using the 2^−ΔΔCt^ comparison threshold method. The primer sequences are shown in Table 1.

### 2.3. Bioinformatics Analysis of CaCP15

The full-length cDNA of *CaCP15* was amplified via PCR using specific primers. We analyzed the molecular weight (MW) and isoelectric point (pI) of CaCP15 protein using the pI/MW program. Multiple sequence alignments and phylogenetic tree analysis were performed using DNAMAN (Lynnon Biosoft, Quebec, Canada) and MEGA5.0. Moreover, the secondary structure and three-dimensional (3D) models of CaCP15 were predicted by SOPMA SECONDARY STRUCTURE PREDICTION METHOD (https://npsa-prabi.ibcp.fr/cgi-bin/npsa_automat.pl?page=npsa_sopma.html, accessed on 17 May 2023) and Protein Homology/analogY Recognition Engine V 2.0 (Phyre2; http://www.sbg.bio.ic.ac.uk/phyre2/html/page.cgi?id=index, accessed on 19 May 2023) [23]. The *cis*-acting elements were predicted in *CaCP15* using PlantCARE (http://bioinformatics.psb.ugent.be/webtools/plantcare/html/, accessed on 23 April 2023).

### 2.4. VIGS and Transient Overexpression Assay of CaCP15

The fragments of the *CaCP15* gene were cloned from pepper line B12 and inserted into the pTRV2 vector, as previously described, and *CaPDS* (phytoene desaturase in pepper, accession number: LOC107861625) served as the positive control [24]. After four weeks, the transcriptional level of *CaCP15* was measured in pTRV2: *CaCP15* and pTRV2 plants. *CaCP15*-silenced and control plants were treated with NaCl and mannitol (300 mM) for the stress experiment.

Agrobacterium GV3101 cells harboring pSN1301-GUS-*CaCP15* or pSN1301-GUS-00 (used as a control) were infiltrated into the leaves of pepper plants at the eight-leaves stage for salt and osmotic assays [22,25].

### 2.5. Physiological Parameters Measurements

Total chlorophyll and malondialdehyde (MDA) contents were measured as previously described [26,27]. The H_2_O_2_ and proline contents were determined according to the modified method by Wang et al. [28]. The activities of the antioxidant enzymes (mutase and peroxidase) in pepper leaves were measured as described by Beauchamp et al. and Ranieri et al. [29,30].

### 2.6. Statistical Analysis

SPSS 22.0 software was used to analyze the data (*p* < 0.05). The histograms were generated using SigmaPlot 14.0. The analyzed data were presented as the means ± standard deviation (SD).

## 3. Results

### 3.1. Identification and Characterization of the CaCP15 Gene

**Figure 1:**
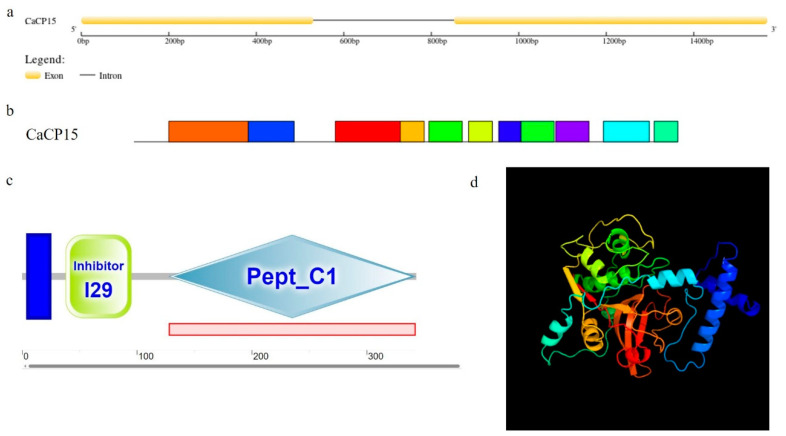
The gene and protein structures of CaCP15. (**a**) The gene structures of *CaCP15*, (**b**) The conserved motifs of CaCP15, (**c**) Two highly conserved domains of CaCP15, (**d**) The three-dimensional models of the CaCP15.

*CaCP15* (LOC107859299) contained a complete open reading fragment (ORF) of 1032 bp, containing 343 amino acids with a theoretical MW of 37.98 kDa and a calculated pI of 5.44. The *CaCP15* was distributed on chromosome 2 (chr2) and predicted to localize in the vacuoles. One intron was found between the nucleotide sites 531–852 (Figure 1a). CaCP15 had eleven consensus motifs (Figure 1b), and the N terminus of CaCP15 contained a transmembrane helix (position F5–T24). Inhibitor 129 (H38-F95) and peptidase_C1 (V128-T342), highly conserved domains, were found in CaCP15 amino acid sequences (Figure 1c). The secondary structure of CaCP15 mainly contained 34.99% of α helices, 15.16% of strands, 6.41% of β turns, and 43.44% of random coils. Thus, the random coil occupied the largest proportion of secondary structures, followed by α helices and extended strands. Moreover, the tertiary structure of CaCP15 was generated using homologous modeling. The 3D models of CaCP15 were based on template c6u7dA (PDB header: plant protein, Chain:A; PDB Molecule:fbsb; PDBTitle: recombinant stem bromelain precursor) (Figure 1d). The composition and location of the secondary structure of the protein were observed distinctly.

### 3.2. Multi-Sequence Alignment and Phylogenetic Analysis of CaCP15

**Figure 2:**
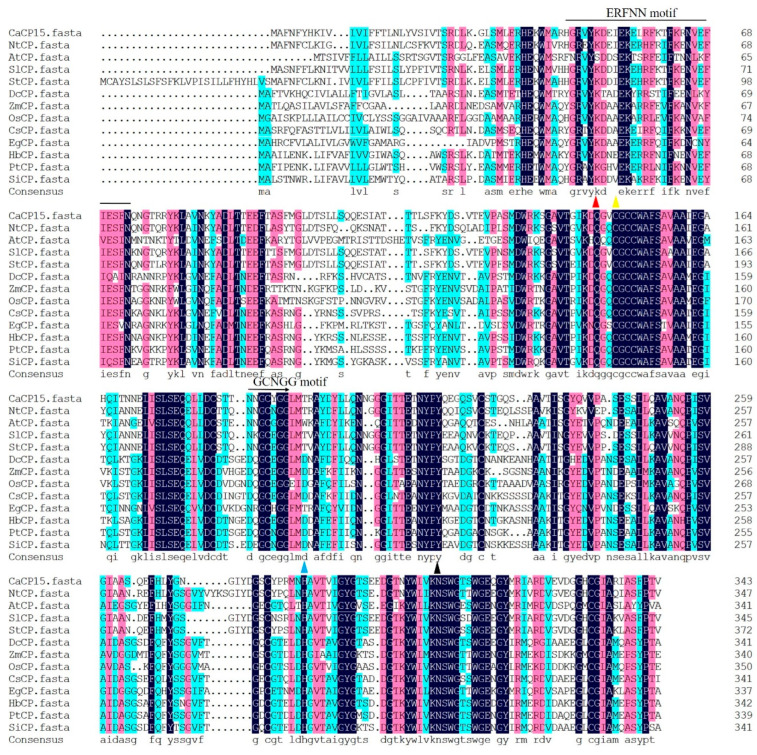
Multiple sequence alignment of CPs. Dark-blue background, pink background, and blue background respectively signified 100%, 75%, and 50% conserved amino acid residues. The single line presented the ERFNIN motif; the arrow presented the GCNGG motif, and the different colors triangles indicated the catalytic triad Cys-, His-, Asn, and Glu-active site residue, respectively.

**Figure 3:**
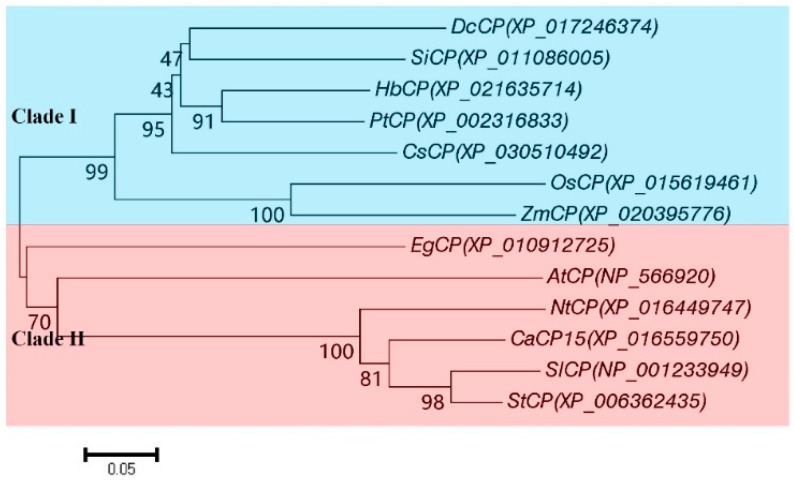
Phylogenetic analysis of CaCP15 and other plants CPs. Ca, *Capsicum annuum* L.; At, *Arabidopsis thaliana*; St, *Solanum tuberosum*; Dc, *Daucus carota*; Sl, *Solanum lycopersicum*; Cs, *Cannabis sativa*; Nt, *Nicotiana tabacum*; Eg, *Elaeis guineensis*; Hb, *Hevea brasiliensis*; Os, *Oryza sativa*; Si, *Sesamum indicum*; Pt, *Populus trichocarpa*; Zm, *Zea mays*.

We previously predicted that CaCP15 belongs to the SAG12 subfamily, containing two highly conserved interspersed ERFNIN motif (position E54–N73) and GCNGG motif (G189–G193). CaCP15 protein contained four conserved CP catalytic triads; Cys (C149), His (H285), Glu residue (Q146), and Asn (N307). Sequence alignment of the CPs from different plants showed high homology (Figure 2). These included NtCP (similarity 76.20%, accession number: XP_016449747.1), SlCP (80.64%, NP_001233949.2), EgCP (56.05%, XP_010912725.1), DcCP (52.29%, XP_017246374.1), HbCP (54.15%, XP_021635714.1), PtCP (54.60%, XP_002316833.3), AtCP (52.79%, NP_566920.1), SiCP (54.02%, XP_011086005.1), OsCP (49.41%, XP_015619461.1), ZmCP (47.23%, XP_020395776.1). A phylogenetic tree showed the evolutionary relationship between the CaCP15 and the other CPs (Figure 3). CaCP15, SlCP, StCP, NtCP, AtCP, and EgCP clustered in the same clade, while, ZmCP, OsCP, CsCP, PtCP, HbCP, SiCP, and DcCP formed a different cluster. CaCP15 was closely related to SlCP and StCP, suggesting they may have a similar function.

### 3.3. Promoter Analysis of CaCP15

**Figure 4:**
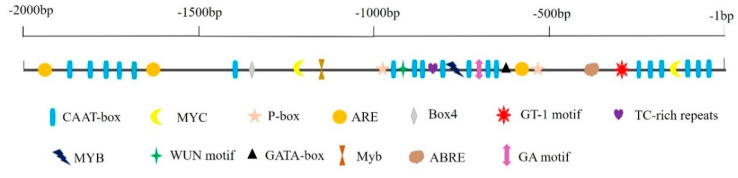
*Cis*-elements prediction in the promoter region of *CaCP15*.

The *cis*-acting regulatory elements of the *CaCP15* promoter were identified to characterize the transcriptional regulation of *CaCP15* (Figure 4). The result revealed the existence of some putative *cis*-acting regulatory elements modulating stress response and defense-related genes in the promoter region. These elements contained one TC-rich repeats (defense and stress responsiveness), one GT-1 *cis*-element (salt-stress response), one MYB (drought- stress related), two MYC (drought-stress related), one Myb (regulated anthocyanin pigment), one ABRE (abscisic acid responsiveness), three ARE (auxin responsiveness), and one WUN motif (wound-responsive). Moreover, we identified 19 CAAT-box (promoter and enhancer regions) and some light-responsive elements (One GATA-box, one GA motif, one Box4, and two P-box). In addition, more than half of all putative *cis*-elements occurred between −1000 to −1 bp within the promoter sequence.

### 3.4. Expression Analysis of CaCP15 in Pepper

**Figure 5:**
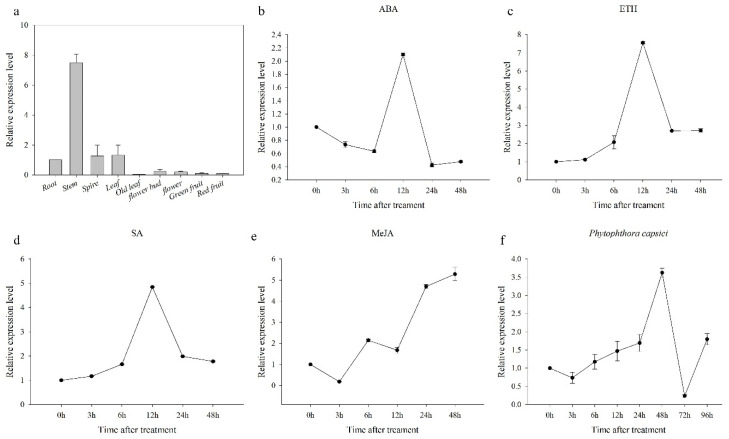
Expression patterns of *CaCP15* in different tissues, responsing to exogenous phytohormones and *phytophthora capsici*. (**a**) Expression of *CaCP15* in different tissues; (**b**) Expression of *CaCP15* under ABA; (**c**) Expression of *CaCP15* under ETH; (**d**) Expression of *CaCP15* under SA; (**e**) Expression of *CaCP15* under MeJA; (**f**), Expression of *CaCP15* under *phytophthora capsici*.

**Figure 6:**
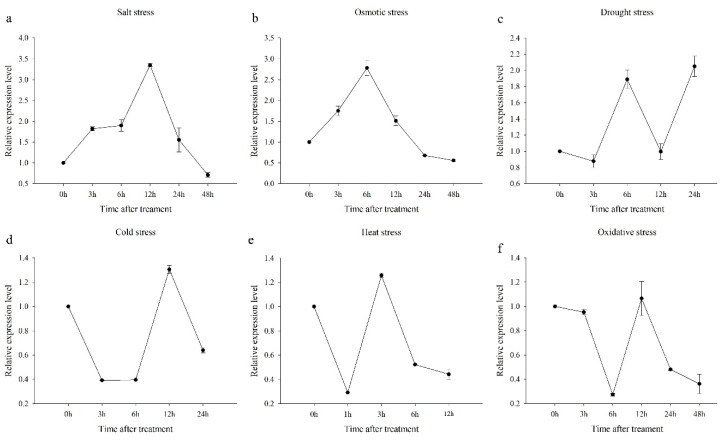
Expression patterns of *CaCP15* under abiotic stresses. (**a**) salt, (**b**) osmotic, (**c**) drought, (**d**) cold, (**e**) heat, and (**f**) oxidative stresses.

To explore the potential functions of *CaCP15*, we analyzed the expression profiles of *CaCP15* in various pepper tissues under various stresses by qRT-PCR (Figure 5 and Figure 6 and [22]). The results showed that *CaCP15* was detected in various tissues. Compared with the roots, the expression level of *CaCP15* in the stems was 8-fold higher, suggesting that *CaCP15* may be involved in stem development (Figure 5a, [22]). The expression of *CaCP15* varied in different leaf development stages. Compared with the roots, *CaCP15* was highly expressed in young and mature leaves than that old leaves (Figure 5a). ABA, ETH, SA, and MeJA were respectively sprayed on the leaves of B12 pepper plants at the six-leaf stage leaves. We found that *CaCP15* was upregulated to varying degrees under different exogenous hormone treatments. ABA slightly decreased the transcription level of *CaCP15* within 6 h of the treatment, but the *CaCP15* transcripts later increased, reaching the peak at 12 h. Interestingly, *CaCP15* was drastically downregulated in the ABA-treated plants at 24 h compared with the control (0 h) (Figure 5b). ETH and SA gradually upregulated *CaCP15* within 12 h of the treatment, resulting in 7.5- and 4.8-fold increments in the *CaCP15* transcripts, respectively, compared to the control. However, the *CaCP15* transcript levels declined rapidly at 48 h (Figure 5c,d). The MeJA treatment slightly downregulated *CaCP15* expression at 3 h and 12 h, reaching the lowest point within the first 3 h, after which *CaCP15* expression was increased, reaching the peak (5-fold) at 48 h post-treatment (Figure 5e). The results indicated that the *CaCP15* gene could be regulated by the four signaling molecules (ABA, ETH, MeJA, and SA).

As shown in Figure 4f, the expression level of *CaCP15* was increased in pepper plants inoculated with *Phytophthora capsicipc*. The *P*. *capsicipc* reduced the expression level of *CaCP15* within the first 3 h after infection but were gradually upregulated the expression upregulated before 48 h post-treatment, reaching the peak of the expression 3.5-fold higher than the control. After that, there was a sharp reduction at 72 h, reaching the lowest expression level. Interestingly, *CaCP15* transcripts were slightly upregulated at 96 h and then reduced to the same expression level at 24 h (Figure 5f). These results suggested that *CaCP15* possibly participated in the pepper resistance to pathogens.

To determine the roles of *CaCP15* in response to salt, osmotic, drought, cold, heat, and oxidative stresses, we artificially altered the growth environment of pepper plants (Figure 6, [22]). For the salt and osmotic stress, pepper plants were soaked in NaCl and mannitol solution, respectively. The *CaCP15* expression was gradually enhanced by NaCl (300 mM) treatment at 3 h and constant until 6 h, followed by an increment that represented the peak expression (to 3.4-fold) at 12 h (Figure 6a). Similarly, mannitol treatment increased the *CaCP15* transcripts, reaching the peak (2.7-fold) at 6 h. However, the expression level of *CaCP15* gradually declined until 48 h post-treatment, at which point the transcript levels were lower than those of the control (Figure 6b). Under drought stress, the transcription level of *CaCP15* was slightly downregulated in the leaves of pepper plants at 3 h but was upregulated from 3 h to 6 h after uprooting the pepper plants. Interestingly, the transcription level of *CaCP15* was rapidly downregulated at 12 h, a same level relative to the control. However, compared to the control (0 h), *CaCP15* expression had a 2.0-fold upregulation under drought stress at 24 h, reaching the peak (Figure 6c). To analyze the abundance of *CaCP15* transcripts under cold and heat stress, we exposed pepper plants to 4 °C and 40 °C in the illumination incubator. Results showed that the transcription level of *CaCP15* showed a downregulation trend at 4 ℃ and 40 °C. As shown in Figure 6d, *CaCP15* expression declined drastically within the first 3 h of the 4 °C treatment and remained constant at 6 h. There was a sharp increase in *CaCP15* expression at 12 h and a decrease at 24 h. In the 40 °C treatment, *CaCP15* expression reduced in the first 1 h and suddenly increased at 3 h. Interestingly, *CaCP15* transcript levels were slightly downregulated from 6 h to 24 h (Figure 6e). Pepper plants were also sprayed with 100 mM H_2_O_2_ to study whether the *CaCP15* gene responded to oxidative stress. Compared with the control (0 h), the transcriptional level of *CaCP15* remained stable at 3 h but was dramatically downregulated at 6 h after treatment, reaching the bottom. Thereafter, the *CaCP15* expression was sharply enhanced at 12 h and gradually downregulated from 24 h to 48 h (Figure 6f). The results showed that the *CaCP15* gene responded positively to these abiotic stresses.

### 3.5. Knockdown of CaCP15 Enhnaces Salt and Osmotic Stress Resistance in Pepper

**Figure 7:**
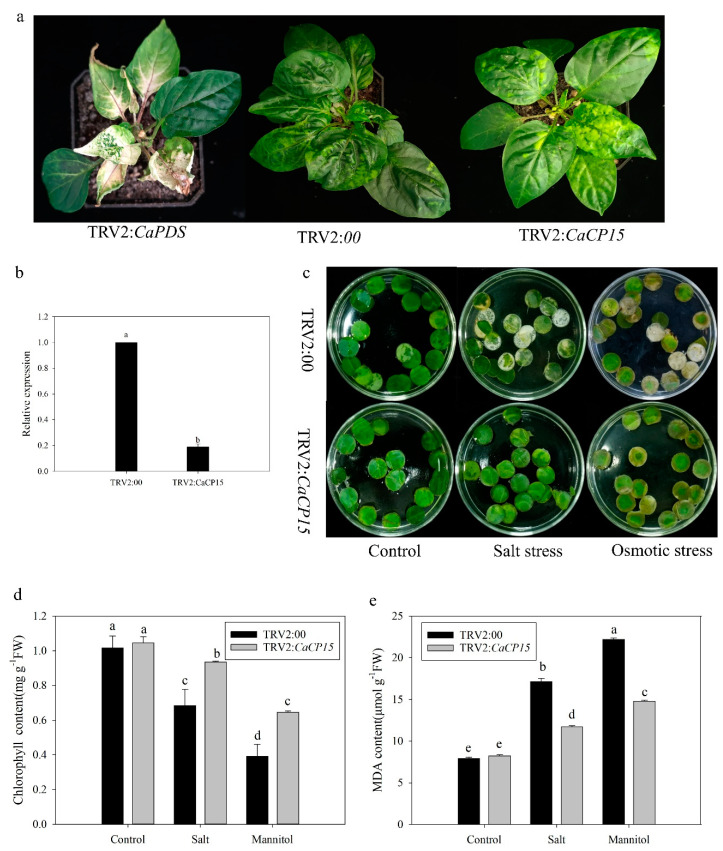
Silencing of *CaCP15* enhances salt and osmotic stress resistance in pepper. (**a**) TRV2-*CaPDS*, TRV2:00, and TRV2-*CaCP15* pepper plants; (**b**) The efficiency of *CaCP15* silencing in leaves; (**c**) The manifestations of *CaCP15*- silenced and control leaves discs to salt and osmotic stresses; (**d**,**e**) Chlorophyll and MDA contents of the *CaCP15*-silenced and control leaf discs in response to 300 mM NaCl and mannitol stresses, respectively. The values are the means ± SE (standard error) of three independent replicates. The letters (a–e) represent significant differences according to Tukey’s test (*p* < 0.05).

*CaCP15* was silenced in pepper by the VIGS technique to verify the function of *CaCP15* under salt and osmotic stress [24]. At two weeks after planting, the B12 pepper plants were infiltrated with *Agrobacterium* cells containing TRV2:00, TRV2:*CaPDS*, and TRV2:*CaCP15* vectors and were subjected to stress treatments after about 45 days of the infiltration. The empty vector TRV2:00 was used as the negative control. Since *CaPDS* silencing caused leaf photobleaching symptoms, the TRV2: *CaPDS* plants were used as the positive controls for detecting VIGS efficiency. As shown in Figure 7a, TRV2: *CaPDS* plants showed obvious leaf photobleaching symptoms, indicating that the VIGS system was successful. Compared with the TRV2:00 plants, TRV2:*CaCP15* plants had no morphological changes after 45 days of inoculation (Figure 7a). Therefore, we measured the expression level of *CaCP15* in the leaves of TRV2:00 and TRV2:*CaCP15* plants by qRT-PCR. The efficiency of *CaCP15* silencing was 80% lower in the *CaCP15*-silenced plants compared with the control, implying that *CaCP15* was successfully silenced by the VIGs assays (Figure 7b). To determine the function of *CaCP15* under salt or osmotic stress, we exposed the leaf discs (1.0 cm in diameter) from the leaves of TRV2:00 and TRV2:*CaCP15* plants to NaCl or mannitol solution (300 mM), with sterile water as the control. After 3 days, the leaf discs of the control plants subjected to salt or osmotic stress exhibited a bleached phenotype compared to those subjected to the control (sterile water), and more obvious than the leaf discs of TRV2: *CaCP15* under stress (Figure 7c). Hence, we measured the chlorophyll content of the leaf discs under different treatments. The chlorophyll content in the TRV2:*CaCP15* and TRV2:00 plants was reduced after treatment, and the leaf discs of the TRV2:00 plants degraded more than those of *CaCP15*-silenced plants discs (Figure 7d). Moreover, we also measured the MDA content, which reflected the degree of leaf damage under stress. As shown in Figure 7e, MDA accumulation was gradually increased in TRV2:*CaCP15* and TRV2:00 plants after NaCl or mannitol treatment, but the MDA content of the control plants was higher than that of TRV2:*CaCP15* plants. These findings proved that *CaCP15* silencing could enhance the salt and osmotic stress resistance in pepper. 

### 3.6. Transient Overexpression of CaCP15 Reduces Salt and Osmotic Stress Resistance in Pepper

**Figure 8:**
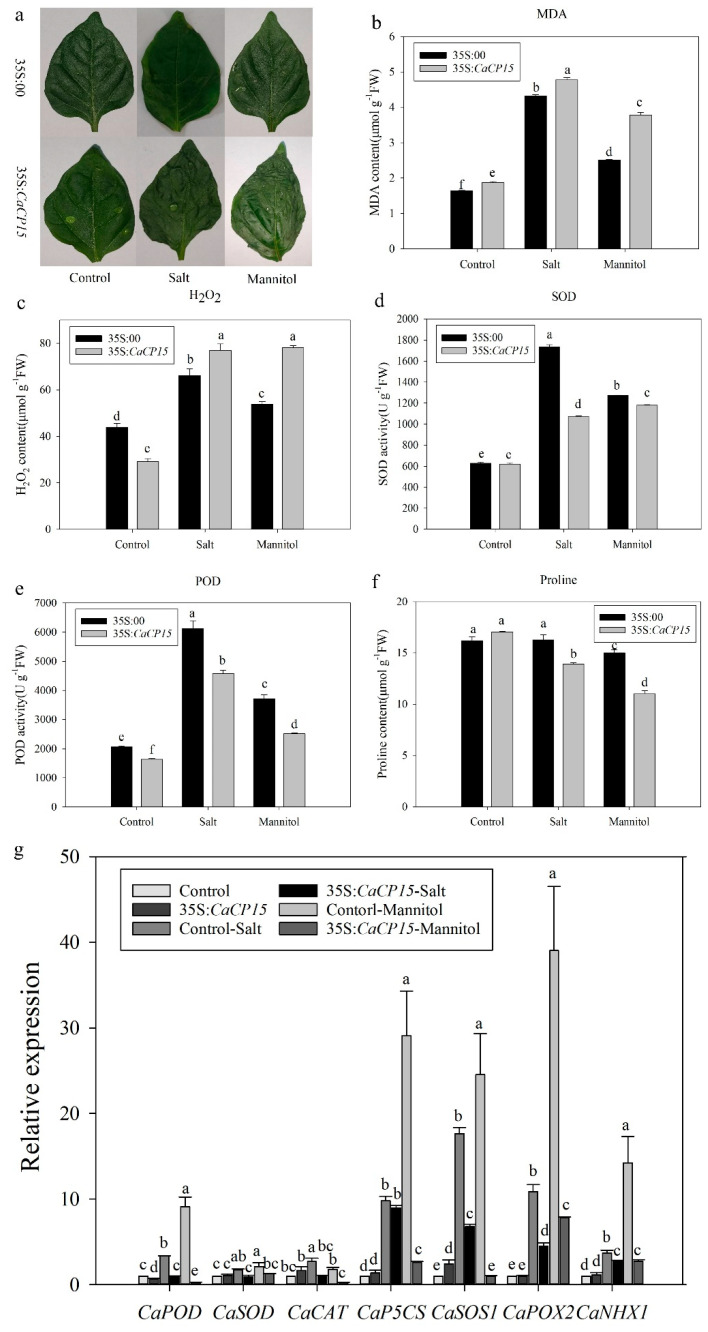
Transient overexpression of *CaCP15* reduces salt and osmotic stress resistance in pepper. (**a**) Phenotypes of pepper leaves under salt or mannitol stress and control; (**b**) MDA and (**c**) H_2_O_2_ contents; (**d**,**e**) SOD and POD enzymes activities, and (**f**) Proline content; (**g**) Expression of antioxidant-related genes and stress-related genes in overexpression and control plants. All the values are the means of three independent replicates ± SE (standard error). Means with different letters represent significantly different according to Tukey’s test (*p* < 0.05).

To further investigate the function of *CaCP15* in salt and osmotic stress tolerance, we overexpressed *CaCP15* in pepper leaves using the 35S:*CaCP15* vector, with taking the 35S:*00* empty vector as the control. The infiltrated plants were treated with NaCl, mannitol, and water (control). After 12 h post-treatment, the leaves of *CaCP15*-overexpressing plants had significantly wilted compared with the 35S:*00* leaves under salt and osmotic stress (Figure 8a). We further measured the physiological indexes related to the ROS system. The leaves of *CaCP15*-overexpressing and control plants showed excessive MDA accumulation, which was higher in the *CaCP15*-overexpressing leaves than in the 35S:*00* plants under stress (Figure 8b). The MDA content in 35S: *CaCP15* plants showed a 51.1% increase compared with 35S:*00* plants under osmotic stress. Similarly, the H_2_O_2_ content was increased in all plants under stress, and the content was markedly higher in CaCP15-overexpressing leaves than in the 35S:*00* plants. The H_2_O_2_ content of the *CaCP15*-overexpressing leaves increased by 16.1% under salt stress and 45.3% under osmotic stress (Figure 8c). In addition, superoxide dismutase (SOD) and peroxidase (POD) activities in 35S: *CaCP15* and 35S:*00* plants were significantly enhanced under salt or osmotic stress compared with the control. However, the accumulation of main ROS-scavenging enzymes in the *CaCP15*-overexpressing leaves was significantly lower than in control leaves under stress. Compared with the control, the activities of SOD in 35S:*CaCP15* plants decreased by 38.0% under salt stress, while the activities of POD reduced by 25.5% under salt stress and 32.3% under osmotic stress (Figure 8d,e). In contrast, the proline content was reduced in response to salt and osmotic stress in all plants. The degree of reduction in the *CaCP15*-overexpression leaves (14.5% under salt and 26.7% under osmotic stress) was more obvious than in 35S:*00* plants (Figure 8f). Furthermore, we also measured the transcriptional level of antioxidant-related genes (*CaPOD*, *CaSOD*, and *CaCAT*) and stress-related genes (*CaNHX1*, *CaP5CS*, *CaPOX2*, and *CaSOS1*) to analyze the function of *CaCP15* in pepper under salt or osmotic stress. As shown in Figure 8g, there was a significant increase 35S:*CaCP15* and 35S:*00* plants under salt or mannitol stress, and the transcriptional levels of these genes were significantly lower in the *CaCP15*-overexpression leaves than in the control plants (Figure 8g). Overall, *CaCP15* overexpression increased the sensibility to salt and osmotic stresses, suggesting that *CaCP15* may play a negative regulatory role in the salt and osmotic stress resistance of pepper.

## 4. Discussion

PLCPs are a functional proteolytic enzyme family involved in plant growth, development, senescence, immune and stress responses [31]. The PLCPs family has complementary and redundant functions, making it difficult to determine the functional importance of a particular PLCP in plants. In this study, we characterized a multiple stress-induced proteolytic enzyme CaCP15. CaCP15 is a member of the SAG12 subfamily, with two typical conserved domains: “ERFNIN” and “GCNGG” motifs. This is consistent with the other members of the SAG12 subfamily [22]. The CPs sequences of other plants also have “ERFNIN” and “GCNGG” conserved regions, demonstrating that the functions of the two domains are important. The evolutionary tree analyses of the CP proteins showed CaCP15 was homologous to NtCP15 and SlCP15 in tobacco and tomato, respectively. A previous study have proved that SlCP15 is one of the immune proteases in tomatoes [32]. *NtCP15* confers resistance to pathogens [33]. Similarly, we verified that *CaCP15* expression was increased after *P*. *capsicipc* treatment. Besides, MeJA and SA applications increased the *CaCP15* transcripts to 5.0-fold compared with the control. MeJA and SA are critical in plant defense against pathogen infection [34,35]. Our results indicated that *CaCP15* might be involved in the resistance against pathogenic bacteria through the MeJA- and SA-dependent signaling pathways in pepper. The tissue expression analysis of *CaCP15* in pepper showed that the transcription level of *CaCP15* was 8-fold higher in the stems than in the roots, suggesting that the gene may play a role in stem development. 

In plants, CPs are involved in salt and osmotic stress responses. For example, the expression levels of *AtRD21A* and *AtRD19A* in *Arabidopsis* were increased under salt stress [12]. The transcription level of *Cyp15a* was increased in pea seedlings treated with NaC1 [13], and the wheat *PLCP* gene (*TaCP*) was upregulated by salt stress [36]. *SPCP2*-overexpressing *Arabidopsis thaliana* had enhanced salt stress resistance [14]. Salinity stress increased the expressions of *CPs* genes (*LOC_Os01g73980*, *LOC_Os02g27030,* and *LOC_Os05g01810*) in rice [37]. The two barley CysProt were involved in drought stress response [38]. Furthermore, *CaCP11* and *CaCP34* participated in salt and mannitol stress resistance of pepper [22,24]. These studies suggest that *CPs* may play important roles in abiotic stress responses in plants. We identified several *cis*-elements in putative promoter regions of *CaCP15*, which could respond to signal molecules and environmental stresses. Interestingly, one GT-1 motif, a *cis*-acting element involved in response to salt stress [39], and one MYB and two MYC drought-stress-related *cis*-acting elements were found in the *CaCP15* promoter [40]. Hence, we used qRT-PCR analysis to verify the function of *CaCP15* under abiotic stress and exogenous plant hormone application. The results revealed that *CaCP15* was regulated by salt and osmotic stress, and its transcription level increased by 3.3-fold at 12 h under NaCl treatment and 2.7-fold at 6 h under mannitol treatment compared with at 0 h. In addition, ABA application upregulated *CaCP15* expression, and the expression level at 12 h was 2.0-fold higher than that at 0 h. Similarly, ETH treatments also enhanced the *CaCP15* expression, and the expression level at 12 h was increased by 7.5-fold compared with at 0 h. Since ABA and ETH signaling pathways are central regulators of abiotic stress responses in plants, we hypothesized that *CaCP15* responded to abiotic stress through the ABA or ETH signaling pathway [41,42,43]. We used VIGs and transient overexpression assay to further verify the function of *CaCP15* in response to salt and osmotic stress. Chlorophyll content can reflect the damage degree of plants under stress [44]. It was found that deletion or overexpression of CPs, such as *AtCEP1* and *HvPAP14*, could induce changes in the expression of photosynthetic genes in plants [45,46]. Thus, chlorophyll content can be affected by CPs in the cytoplasm [47]. Compared with control plants, the total chlorophyll content in the *CaCP15*-silenced leaves showed a 36.7% and 64.6% increase after NaCl and mannitol treatments, respectively. ROS-induced lipid peroxidation is an internal indicator of ROS damage, reflected by the MDA content [48]. MDA is generally used to evaluate the degree of ROS-mediated lipid peroxidation in plants under high salt stress [48]. The MDA content in the *CaCP15*-silenced leaves was lower than control plants after the treatments, and the *CaCP15*-silenced leaves showed a 31.6% reduction under salt stress and a 33.4% reduction under osmotic stress. However, the transiently overexpressing-*CaCP15* leaves showed a 51.1% increase in the MDA content and had 43.3% higher H_2_O_2_ contents than the controls control under osmotic stress. Salt stress also slightly increased the MDA and H_2_O_2_ contents in the 35S:*CaCP15* leaves compared to the 35S:*00* plants. H_2_O_2_ is a product of ROS [49]. Thus, these results showed that *CaCP15* might play a negative role in the abiotic stress response of pepper by clearing ROS accumulation. We also observed the variation in the activities of the major ROS scavenging enzymes (SOD and POD) in the transiently overexpressing-*CaCP15* plants experiment with significant differences before and after treatments. The SOD and POD activities of the *CaCP15*- overexpression plants were significantly lower than those of the control. The antioxidant enzyme system and enzyme encoding genes (*CaPOD*, *CaSOD*, and *CaCAT*) were activated under stress conditions to protect pepper from the injuries caused by stress [50]. In our study, the stress treatments reduced the expression of *CaPOD*, *CaSOD*, and *CaCAT* in the *CaCP15*-overexpression pepper plants. Salt stress significantly improved the activities of the antioxidant enzymes to decompose H_2_O_2,_ a product of ROS, suggesting that ROS-scavenging plays an important role in salt tolerance mechanism [51]. These results showed that *CaCP15* overexpression reduced the stress resistance of pepper by reducing the ROS scavenging enzymes activities. 

Proline protects against osmotic stress, and *NtP5CS1* is involved in proline biosynthesis under salt stress [52]. The proline content and the expression of *CaP5CS* were lower in the *CaCP15*-overexpressing leaves than in control under stress. *CaCP15* increased the sensitivity of plants to salt and osmotic stress. Moreover, the stress response genes, such as *SOS*, *NHX1*, *P5CS*, etc., could be activated under stress [53,54,55,56]. The transcription levels of *NtSOS1* and *NtNHX1* were significantly increased in *AlSRG1* transgenic tobacco under salt or osmotic stress, increasing their abiotic stress resistance [57]. *ZmMKK4* regulated osmotic stress response in transgenic tobacco by ROS-scavenging, and *NtPOX1* was upregulated in the *ZmMKK4*-overexpressing plants [58]. In our study, the expression of *CaSOS1*, *CaPOX2*, and *CaNHX1* in *CaCP15*-overexpression pepper leaves was reduced under stress compared to the control leaves, showing that *CaCP15* overexpression enhanced the sensitivity of pepper to salt and osmotic stress.

## 5. Conclusions

In conclusion, CaCP15 is a SAG12 protein containing two highly conserved domains. The expression profile revealed that *CaCP15* was associated with the development of pepper stems and was involved in abiotic and biotic stress responses. *CaCP15* silencing in pepper enhanced salt and osmotic stress resistance. Contrarily, transient overexpression of *CaCP15* reduced salt and osmotic stress resistance by decreasing the antioxidant enzyme activities and negatively regulating the stress-related genes. In summary, *CaCP15* may negatively regulate salt and osmotic stress resistance in pepper. This study demonstrates the molecular and physiological responses of *CaCP15* to salt and osmotic restress in plant. Our future studies will focus on determining the factors the interacting with *CaCP15* under salt and osmotic stress to understand the regulatory pathways and mechanisms related to abiotic stress for breeding stress-resistant pepper varieties.

## Figures and Tables

**Table 1:** Primer sequences in this study.

Primers	Sequence (5′–3′)
Primer sequences of VIGS	
TRV2-*CaCP15*F	GCTCTAGAACCAGCAAGTGAGTCGTCAT
TRV2-*CaCP15*R	CGGGATCCCTTCATGAATCTTCAATTACTAGCT
*CaPDS*F	TGTTGTCAAAACTCCAAGGTCTGTA
*CaPDS*R	TTTCTCCCACTTGGTTCACTCTTGT
Primer sequences of ORF	
*CaCP15*	GGTACCATGGCATTCAATTTTTACCACAAAA
*CaCP15*	GGATCCTCAAACAGTTGGGAAAGAAGC
Quantitative real-time PCR	
*CaCP15*-F	TGGCAGAGCATGGGAAAGTA
*CaCP15*-R	CGTGCCCAAATACATAGCCC
*CaUBI3*-F	TGTCCATCTGCTCTCTGTTG
Ca*UBI3*-R	CACCCCAAGCACAATAAGAC
*CaPOD*-F	AACAGGGAAACCCGAATGGG
*CaPOD*-R	TTTGGTGCAGCCCTCTTCTC
*CaSOD*-F	GAGAACCGTCATGCTGGTGA
*CaSOD*-R	GAGAGGAATCTGCTCGTCGG
*CaCAT*-F	AAGCAGGCTGGGGAGAGATA
*CaCAT*-R	CATGAGTGACTCGGGGATCG
*CaP5CS*-F	ATTCTGCTGATCCTGCTCGG
*CaP5CS*-R	CCCGAATCTGCTCACACAGT
*CaPOX2*-F	ACCCAACGATAACTCAGCCA
*CaPOX2*-R	AGTTGGCTGTTCTTGCATCG
*CaSOS1*-F	ACTGGAGCTGGTCAACATCA
*CaSOS1*-R	AGCTCCCCAGTTAAAGGTCC
*CaNHX1*-F	AGGCAGTCGAGTACAGTGTC
*CaNHX1*-R	ATGGGGCGCATGAATGAATC

## Data Availability

Not applicable.
